# Ticks and Tick-Borne Pathogens in Domestic Animals, Wild Pigs, and Off-Host Environmental Sampling in Guam, USA

**DOI:** 10.3389/fvets.2021.803424

**Published:** 2022-01-11

**Authors:** Genevieve V. Weaver, Neil Anderson, Kayla Garrett, Alec T. Thompson, Michael J. Yabsley

**Affiliations:** ^1^Wise Owl Animal Hospital, Micronesian Exotic Specialty Services, Tamuning, GU, United States; ^2^The Royal (Dick) School of Veterinary Studies and the Roslin Institute, University of Edinburgh, Edinburgh, United Kingdom; ^3^Southeastern Cooperative Wildlife Disease Study, College of Veterinary Medicine, University of Georgia, Athens, GA, United States; ^4^Warnell School of Forestry and Natural Resources, University of Georgia, Athens, GA, United States

**Keywords:** Guam, ticks, tick-borne disease, tick-borne pathogen, domestic animals, dogs *(Canis familiaris)*, wild pigs *(Sus scrofa)*, sentinels

## Abstract

**Background:** Guam, a United States of America (USA) island territory in the Pacific Ocean, is known to have large populations of ticks; however, it is unclear what the risk is to wildlife and humans living on the island. Dog (*Canis familiaris*), cat (*Felis catus*), and wild pig (*Sus scrofa*) sentinels were examined for ticks, and environmental sampling was conducted to determine the ticks present in Guam and the prevalence of tick-borne pathogens in hosts.

**Methods and Results:** From March 2019-November 2020, ticks were collected from environmental sampling, dogs, cats, and wild pigs. Blood samples were also taken from a subset of animals. A total of 99 ticks were collected from 27 environmental samples and all were *Rhipicephalus sanguineus*, the brown dog tick. Most ticks were collected during the dry season with an overall sampling success rate of 63% (95% CI: 42.4–80.6). 6,614 dogs were examined, and 12.6% (95% CI: 11.8–13.4) were infested with at least one tick. One thousand one hundred twelve cats were examined, and six (0.54%; 95% CI: 0.20–1.1) were found with ticks. Sixty-four wild pigs were examined and 17.2% (95% CI: 9.5–27.8) had ticks. In total, 1,956 ticks were collected and 97.4% of ticks were *R. sanguineus*. A subset of *R. sanguineus* were determined to be the tropical lineage. The other tick species found were *Rhipicephalus microplus* (0.77%), *Amblyomma breviscutatum* (0.77 %), and a *Haemaphysalis* sp. (0.51%). Blood samples from 136 dogs, four cats, and 64 wild pigs were tested using polymerase chain reaction (PCR) and DNA sequencing methods. Five different tick-borne pathogens with the following prevalences were found in dogs: *Anaplasma phagocytophilum* 5.9% (95% CI: 2.6–11.3); *Anaplasma platys* 19.1% (95% CI: 12.9–26.7); *Babesia canis vogeli* 8.8% (95% CI: 4.6–14.9); *Ehrlichia canis* 12.5% (95% CI: 7.5–19.3); *Hepatozoon canis* 14.7% (95% CI: 9.2–28.8). *E. canis* was detected in one cat, and no tick-borne pathogens were detected in wild pigs. Overall, 43.4% (95% CI: 34.9–52.1) of dogs had at least one tick-borne pathogen. Serological testing for antibodies against *Ehrlichia* spp. and *Anaplasma* spp. showed prevalences of 14.7% (95% CI: 9.2–28.8) and 31.6% (95% CI: 23.9–40), respectively.

**Conclusion:** Four different tick species were found in Guam to include a *Haemaphysalis* sp., which is a previously unreported genus for Guam. Dogs with ticks have a high prevalence of tick-borne pathogens which makes them useful sentinels.

## Introduction

Vector-borne diseases are one of the greatest threats to human and animal health. After mosquitoes, ticks are the second most significant vector (1). Ticks are critical vectors of deadly pathogens worldwide and a variety of bacterial, protozoal, filarial, and viral pathogens have been identified in the microbial flora of ticks. Some examples of major tick-borne diseases (TBD) include babesiosis, theileriosis, cytauxzoonosis, hepatozoonosis, acanthocheilonemiasis, anaplasmosis, ehrlichiosis, typhus, spotted fevers, Lyme borreliosis, tularemia, bartonellosis, and viral encephalitis (2–4). Tick-borne disease provides good examples of the intersection between human, domestic animal, and wildlife health. Many of the estimated 900 different species of ticks are generalists and can easily attach to other hosts when their preferred host is unavailable (5). Similarly, tick-borne pathogens (TBP) may have their reservoir in one vertebrate species but incidentally infect another species (3). Tick-borne pathogens can cause morbidity in domestic animals and humans. Each year in the US alone, there are estimated to be about 300,000 cases of Lyme disease in humans (6) as well as increasing cases of other TBD such as ehrlichiosis and anaplasmosis (7). Tick-borne disease in cattle such as anaplasmosis can result in an 80% economic loss in some parts of the world (8). Ticks can transmit disease to wildlife (9). For example, in one study in southern Africa, domestic animals were found to be essential in the transmission of ticks and TBP to wildlife (10).

Infectious disease in general can influence the conservation of vulnerable species. The impact of TBD specifically in wildlife is not completely understood; however, many studies over the last decade have found evidence of TBP in wildlife populations worldwide to include: *Rickettsia* spp. and *Anaplasma ovis* in ticks from Cypriot mouflon (*Ovis orientalis ophion*) (11); *Babesia* spp. in gray kangaroos (*Macropus giganteus*) (12); *Theileria, Anaplasma, Ehrlichia*, and *Babesia* genera in wildlife in South Africa (13) and in Tanzania (14); *Borrelia burgdorferi* in brown bears (*Ursus arctos*) in Sweden (15); *Borrelia* spp. and *Rickettsia spp*. in Mongolian small mammals (16); novel species of *Anaplasma* and *Ehrlichia* organisms in Amazonian wildlife (17); and, a variety of TBP in raccoons (*Procyon lotor*), opossums (*Didelphis virginiana*), feral swine (*Sus scrofa*), and white-tailed deer (*Odocoileus virginianus*) in the USA (18).

As ticks are ubiquitous, TBD has consequently had a global impact on human and animal health. Guam (13° 26' 39.4944” N and 144° 47' 37.4352” E) isolated within Oceania has not been spared the impact of ticks. Guam is an island territory of the US located in Micronesia in the Western Pacific Ocean ~2,600 km east of the Philippine Islands and 6,370 km southwest of the Hawaiian Islands (19). It is a tropical island with two seasons (dry and rainy), moderate to high humidity, and consistent year-round warm temperatures averaging 29°C with a range between 21 and 32°C. The dry season is typically from January through June while July through December is the rainy season (20). The human population includes over 160,000 residents to include about 10,000 rotating active-duty military personnel with their families (21). In addition, over one million tourists per year visit the island. This results in frequent travel of people and animals into and out of Guam. The international population living in Guam frequently travels to many parts of Asia and could easily bring in “hitchhiker” ticks in luggage or on their person. An example of tick movement occurred in Connecticut in the US where *Hyalomma truncatum* from southern Africa was found on a person with recent travel history (22). In another case, the Asian longhorned tick (*Haemaphysalis longicornis*) became established in the US very recently after an accidental introduction from a traveler (23).

There are five known species of ticks that have been found in Guam: four hard-bodied ticks of the family Ixodidae and one soft body tick of the family Argasidae (24). These include: the swine tick, *Amblyomma breviscutatum*, the soft bodied tick found on migratory seabirds, *Carios capensis* (previously *Ornithodoros capensis*) (24), the mangrove monitor (*Varanus indicus*) lizard tick, *Amblyomma squamosum* first recorded by Kohls (25), the southern cattle tick, *Rhipicephalus microplus*, a common worldwide parasite of livestock (26), and the most abundant tick species seen on dogs, cats, and humans in Guam, the brown dog tick (*Rhipicephalus sanguineus*) (24).

Several studies on ticks and TBP have previously been conducted in Guam (Supplementary Table 1). At least three of the five identified species of ticks in Guam carry TBP such as *Anaplasma* spp., *Ehrlichia* spp., and *Babesia* spp. (27, 28); however, a full characterization of the diseases has not been conducted. Mehrpad et al. (29) found heavy burdens of ticks in Philippine deer (*Rusa marianna*) and found deer that were positive for *Anaplasma platys, A. marginale*, and the zoonotic *A. phagocytophilum*. Johnson et al. (28) identified two species of ticks and found the vectors positive for *A. platys, A. marginale, Coxiella burnetii* (the agent of Q fever), *Babesia canis vogeli*, and *H. canis*. Cleveland et al. (27) collected ticks from wild pigs and Philippine deer and found ticks positive for *Rickettsia* spp., of which certain species can be zoonotic.

Tick-borne diseases are a threat to Guam's domestic animals, humans, and wildlife populations, and include the small and vulnerable populations of native and migratory birds and one bat species. At least two of the tick species present in Guam are capable of hosting pathogens such as the agents of human ehrlichiosis and spotted fevers which can cause severe autoimmune conditions and organ failure (30). *Anaplasma phagocytophilum* has been found in ticks in Guam (29), and both birds and bats have the potential to become infected with this pathogen (31–33). The Guam rail (*Gallirallus owstoni*) is highly vulnerable and was only recently upgraded from extinct in the wild to critically endangered thanks to intensive breeding and reintroduction efforts over several decades (34). The Mariana fruit bat (*Pteropus mariannus*) does not fare much better with a critically endangered status in Guam due to only a few colonies of <50 individuals (35). In addition, Guam is a seasonal home to over 100 species of migrating sea birds with either transient or semi-permanent colonies as Micronesia is part of an oceanic migratory route (24).

Guam's biodiversity has been severely and, in many cases, irreversibly diminished mainly due to invasive species such as wild pigs, dogs, the brown tree snake, and rodents where numbers of individuals can be in the tens of thousands and even millions as in the case of the brown tree snake. Although several of these species have caused irreparable damage to Guam's wildlife, they do afford the opportunity for sentinel surveillance. Looking at sentinel animals can be an important step in understanding the impact of diseases, especially vector-borne diseases. Sentinels such as dogs, cats, or wild pigs have shown to be effective in extrapolating disease threats to native wildlife, humans, and other domestic animals (36), and sentinels are useful for determining abundance and species of ticks (37). Wild pigs, as a larger sentinel host, have more expansive home ranges, travel longer distances, and may visit more diverse habitats than smaller species. This increases their likelihood of acquiring a variety of pathogens (38). Wild pigs are used as sentinels for tuberculosis in New Zealand (39), and in the Pantanal in Brazil, the tick species *Amblyomma sculptum* from wild pigs is used to extrapolate disease risk in humans (40). Sentinels can often provide a better sampling method compared with dragging or flagging in areas where vegetation is variable, and the abundance of ticks is not uniformly distributed. Sentinel monitoring can detect newly introduced tick species and changes in ranges of a species (37). Finally, sampling of sentinel species such as domestic dogs and feral pigs is easier, both from a logistic and ethical standpoint, compared with most wildlife and humans (41).

With the importance of ticks and their realized and potential impacts on animal and human health, this project was conducted to improve our understanding of ticks and TBD in Guam. The specific goals of this research project are to identify tick species observed in Guam using samples collected from the environment (off-host) and animal hosts (dogs, cats, and wild pigs). We also tested hosts for selected TBP.

## Materials and Methods

Sample collection was divided into off-host environmental sampling and animal sampling. Environmental sampling locations were selected to include a variety of vegetation types and different seasons; however, sampling sites and timing was by convenience, and not randomized. All attempts were made to ensure that the sampling procedure was uniform in distance and time. Sampling of animals was also convenience sampled. All ticks collected from the environment and animals were preserved in 95% ethanol. Ticks were kept at room temperature until shipping to the Southeastern Cooperative Wildlife Disease Study (SCWDS) at the University of Georgia (Athens, GA) for identification.

### Environmental Tick Sample Collection

Environmental sampling was conducted from May 2019 to January 2020. Due to various limitations, the areas selected for sampling were based on ability to access the land area, favorable weather (no rain), and the availability of an experienced guide when needed to navigate the locations. The date, season, Global Positioning System (GPS) coordinates for the start of the sampling event, vegetation type, and number of ticks collected were recorded for every location sampled. Three of the five major landcover classes (rangeland, forest, and urban vegetation) of Guam as defined by Liu and Fischer (42) were sampled (Supplementary Table 2).

Ticks were flagged using a method that would sample leaf-litter vegetation and taller grasses (43, 44). A piece of white cotton flannel cloth ~0.5 m in width and 1 m in length with an attached 0.6-m wooden dowel and twine was used to collect ticks. The flag was dragged behind and to the sides of the researcher through vegetation elevating the flag up to 1 m as needed depending on the vegetation type. In total, each sampling event occurred for ~1 h and covered a 3 km roundtrip in distance (1.5 km each way). Flagging/dragging was stopped every 20 min (three times for each sampling event) to check the cloth for the presence of ticks by meticulously scanning both sides.

### Domestic Animal Sample Collection

Samples of ticks and/or blood were collected from dogs or cats that were presented to a veterinary hospital in Guam. The inclusion criteria for this study were: (1) animals must have had at least one tick on them at the time of presentation, (2) the owner consented to be part of the study, and (3) for animals with a matching blood sample, they were already undergoing venipuncture for routine or illness related diagnostic testing. Data recorded for the individuals included in the study were physical address, season/date of collection, age, sex, species, tick burden, medical diagnosis based on laboratory and/or clinical exam findings, results of antibody testing (*Ehrlichia* and *Anaplasma* only), and results of PCR testing. Each sample was given an identifying number for usage for submission to the laboratory to anonymize the samples. The original data sheet with the above information was kept only by the lead author. Any identifying information already existed in the electronic medical record database system (EzyVet), which is password protected and administration rights are given only to the veterinarians of the clinic, one of which is the lead author.

Ticks were collected from March 2019 through November 2020 from dogs and cats. For dogs and cats, ticks were collected from different anatomical locations (most commonly ears, interdigital areas, axilla, ventral abdomen, dorsum, and face) where tick clusters were found.

Blood samples were collected from dogs and cats from July 2019 to November 2020. The samples were collected from both pets and stray animals that came to the hospital for routine or illness-related diagnostics and treatment. Animals were designated sick or healthy based on the presence or absence of clinical and/or laboratory abnormalities. Animals were designated as sick if they had one or more of the following abnormalities not attributable to another illness: fever, lethargy, anorexia, thrombocytopenia (<150,000 platelets/μl), anemia (<35% hematocrit), or any evidence of a potential coagulopathy such as petechiae, epistaxis, seizures, hematuria, melena, hematochezia, and hematemesis. Animals were safely restrained by Certified Veterinary Technicians and a blood sample was obtained from the jugular, cephalic, or saphenous veins. Blood was placed in both an ethylenediaminetetraacetic acid (EDTA) tube and in a tube without additive or with a serum separator to obtain serum after centrifugation. A small portion from the clinical sample obtained (~0.5–2 ml) of both the whole blood and serum sample was used for this study. Whole blood and sera were frozen at −20°C until shipping SCWDS for testing.

### Wild Pig Sample Collection

Samples were only able to be collected opportunistically from wild pigs from November 2019 to January 2020. Trapping is done throughout the year by the USDA Wildlife Services across the island based on requests by private landowners or local mayors. All pigs were humanely euthanized by gunshot. Pigs were carefully checked for ticks with special emphasis on the ears, axilla, and interdigital areas.

Blood samples were taken as part of the USDA's routine testing of captured wild pigs in Guam. Serum and whole blood from wild pigs trapped and euthanized from November 2019 to January 2020 were provided for testing. Samples were obtained opportunistically based on the presence of successfully trapped pigs and depending on the location that the USDA teams were currently working. Blood samples (2 ml) were taken from the jugular vein and immediately placed in a plastic EDTA tube. The sample was kept on ice for 2–4 h before storage at −20°C.

### In-house Diagnostic Laboratory Testing of Dogs

Whole blood or serum was tested for the presence of antibodies against *Anaplasma* spp. (*A. platys* and *A. phagocytophilum*) and *Ehrlichia* spp. (*E. canis, E. ewingii*, and *E. chaffeensis*) using the VETSCAN^®^ Canine *Anaplasma* Rapid Test and VETSCAN^®^ Canine *Ehrlichia* Rapid Test (Zoetis, Inc.).

### Reference Diagnostic Laboratory Testing

All ticks were identified to genus or species based on morphology and classified by life stage and sex using identification keys from Keirans and Litwak (45) and Walker et al. (46). Any ticks that could not be identified due to damage were characterized using sequence analysis of the 16s rRNA gene as described by Lv et al. (47). A subset of *R. sanguineus* ticks were submitted for sequencing to determine lineage using a fragment of the 16S rRNA gene as described by Lv et al. (47).

Molecular assays were used to screen for infections with *Babesia* spp., *E. canis, Hepatozoon* spp., *A. platys*, and *A. phagocytophilum*. DNA was extracted from 100 microliters of whole blood using the DNAeasy Blood and Tissue kit, (Qiagen, Hilden, Germany). PCR was conducted using a BioRad DNA Engine Peltier Thermal Cycler (Bio-Rad Laboratories Incorporated, Foster City, CA) and published protocols (Supplementary Table 3). Additional details on laboratory methods are provided in Garrett et al. (48).

### Statistical Analysis

IBM SPSS^®^ Statistics was used for all statistical analysis. All proportions were determined along with 95% confidence interval under the non-parametric testing category using the Clopper Pearson Exact test with a 95% confidence level. A binary logistic regression univariable analysis was conducted at a 95% confidence level for the categorical variables assessed with presence of ticks (dependent variable) included season (dry/rainy) and vegetation landcover (rangeland/forest since urban only accounted for four sites). A binary logistic regression with the same confidence level was also done to assess any association between TBP prevalence and other variables. Categorical variables assessed with each positive pathogen status (dependent variable) were season and health status. A univariable analysis was conducted on both season and health status with the dependent variable separately for each of the five pathogens. From the SPSS output of the logistic regression models, beta coefficient, standard error, *p*-value, exp (b) (odds ratio-OR) and 95% confidence interval were recorded and evaluated for any associations among the variables. A *p*-value of < 0.05 was considered statistically significant.

### Ethical Approval

This animal study was approved by the Royal (Dick) School of Veterinary Studies Veterinary Ethical Review Committee (VERC) of the University of Edinburgh for animal usage in clinical research with VERC Reference number 89.19. Written informed consent was obtained from the owners for the participation of their animals in this study.

## Results

### Environmental Tick Sampling

A total of 27 different locations of the three major vegetation landcover class were sampled (forest, rangeland, and urban) across the island of Guam. In total, 99 ticks were collected during 17 sampling events, a 63% success rate (95% CI: 42.4–80.6). Sixteen locations recovered ticks identified as *R. sanguineus* larvae while one location recovered an adult male *R. sanguineus*. The greatest number of sampling events and the most successful sampling events occurred in the dry season with a success in 11 of the 14 sampling events. while the rainy season resulted in only 6 of 13 sampling events being successful (Table 1). The most common landcover type that was sampled was the rangeland which included savanna complex vegetation (mostly sword grass over volcanic fields and coastal strand vegetation found along the beaches). Ticks were recovered in 8 of the 12 sampling events of this landcover type. The second greatest number of sites were included in the forest landcover category and was successful in 6 of 11 sampling events, The least sampled landcover class was urban but was successful in three of the four sampling events.

**Table 1:** Number of positive (ticks collected) environmental sampling events by different vegetation landcover classes and seasons, *N* = 27.

	**Rangeland (*N =* 12)**	**Forest (*N =* 11)**	**Urban (*N =* 4)**	**Overall positive**
Dry (*N =* 14)	6	4	1	11
Rainy (*N =* 13)	2	2	2	6
Overall positive	8	6	3	17 (63%: 42.4–80.6)

A binary logistic regression univariable analysis was used to determine if there was any relationship between a successful sampling event and season or vegetation landcover class. This analysis showed that the prevalence of positive drags was significantly higher in the dry season compared to the rainy season (*p* = 0.032, odds ratio 8.49 (95% CI: 1.19–60.29)]. No difference was noted for rangeland vs. forest (*p* = 0.73, odds ratio 1.4 (95% CI: 0.21–9.51).

### Animal Tick Surveillance

In total, 1,956 ticks were collected and identified from 190 dogs, 11 wild pigs, and six cats. Most ticks (1,906, 97.4%) were identified as *R. sanguineus*. Thirty *R. sanguineus* were sequence confirmed to be tropical lineage (all were 100% identical to each other and 100% identical to a *R. sanguineus* (AY883868) specimen collected from southeast Asia). Fewer numbers of ticks were identified as *R. microplus* (15, 0.77%), *A. breviscutatum* (15, 0.77%), unidentified *Rhipicephalus* sp. (10, 0.51%), and a *Haemaphysalis* sp. (10, 0.51%) (Table 2).

**Table 2:** Species and abundance of ticks from dog, wild pig, and cat sentinels.

**Quantity and species of ticks (number of different sentinels)**	**Cat**	**Wild pig**	**Dog**	**Total**
*Amblyomma breviscutatum*	2 (1)	13 (11)	0	15 (12)
*Haemaphysalis* sp.	6 (1)	0	4 (2)	10 (3)
*Rhipicephalus microplus*	1 (1)	0	14 (3)	15 (4)
*Rhipicephalus sanguineus*	5 (2)	0	1,901(185)	1,906 (187)
*Rhipicephalus* spp.	10 (1)	0	0	10 (1)
Total number of ticks collected	24 (6)	13 (11)	1,919 (190)	1,956 (207)

Dogs were the most frequent sentinels sampled, and the ones contributing the greatest quantities of ticks. In total, 6,614 dogs were seen at the local animal hospital during the research project, and 833 (12.6%; 95% CI: 11.8–13.4) were infested with at least one tick. In contrast, 1,112 cats were seen during the same period, and only six (0.54%; 95% CI: 0.20–1.1) were infested with at least one tick. Sixty-four wild pigs were captured and 11 (17.2%; 95% CI: 9.5–27.8) were infested with at least one tick. Of ticks found on dogs, 99% (1,901/1,919) were *R. sanguineus*. For ticks from wild pigs, 100% were identified as *A. breviscutatum*. Although cats with ticks present were infrequent, the six cats sampled had four different species of ticks including one each with *A. breviscutatum, Haemaphysalis* sp., and *R. microplus*, and two cats with *R. sanguineus*. Because cats are an unusual host for *R. microplus*, one specimen was sequence confirmed using the same lineage PCR protocol for *R. sanguineus* (100% identical to an *R. microplus* (KM246883) collected from Malaysia).

### Animal Blood Tick-Borne Pathogens Testing

Serum and whole blood samples were obtained from 136 dogs, 64 wild pigs, and four cats. For wild pigs, whole blood was tested for the presence of *Babesia* spp. and *A. phagocytophilum*. All samples were negative. For cats, only four were tested for all TBP, and one sample was positive for *E. canis*. This cat had one *R. microplus* tick attached and was excluded from the statistical analysis because of the small sample size.

The prevalence and confidence intervals for the following TBP found in dogs are shown in Table 3. Antibodies against *Anaplasma* spp. was the most common positive test with almost 1/3 of samples testing positive. For PCR testing, *A. phagocytophilum, A. platys, B. canis vogeli, E. canis, H. canis* were detected with *A. platys* being the most common TBP found with a prevalence of 19.1%. Overall, 43.4% (59/136; 95% CI: 34.9–52.1) of dogs had at least one TBP (Table 4) while almost 15% of dogs had co-infections with two or more TBP.

**Table 3:** Overall prevalence for each tick-borne pathogen found in the blood samples of dogs via molecular and antibody testing (*Ehrlichia* spp. and *Anaplasma* spp. only), *N* = 136.

**Tick-borne pathogen**	**Positive**	**Prevalence (%)**	**95% CI**
*Anaplasma phagocytophilum*	8	5.9	2.6–11.3
*Anaplasma platys*	26	19.1	12.9–26.7
*Babesia canis vogeli*	12	8.8	4.6–14.9
*Ehrlichia canis*	17	12.5	7.5–19.3
*Hepatozoon canis*	20	14.7	9.2–21.8
*Ehrlichia* spp. antibody	20	14.7	9.2–21.8
*Anaplasma* spp. antibody	43	31.6	23.9–40.1
Both *Ehrlichia* spp. antibody and PCR	7	5.1	21.1–10.3
Both *Anaplasma* spp. antibody and PCR	14	10.3	5.7–16.7

**Table 4:** Prevalence of tick–borne pathogens in dogs (molecular testing only).

**Number of TBP per dog (*N =* 136)**	**Positive**	**Prevalence (%)**	**95% CI**
One pathogen	39	28.7	21.3–37.1
*A. phagocytophilum*	2		
*A.platys*	11		
*B. canis vogeli*	8		
*E. canis*	8		
*H. canis*	10		
Two pathogens	16	11.8	6.9–18.4
*A. platys/A. phagocytophilum*	2		
*A. platys/B. canis vogeli*	2		
*A. platys/E. canis*	4		
*A. platys/H. canis*	3		
*E. canis/H. canis*	4		
*H. canis/A. phagocytophilum*	1		
Three pathogens	4	2.9	0.8–7.4
*A. platys/A. phagocytophilum/B. canis*	2		
*A. platys/A. phagocytophilum/H. canis*	1		
*A. platys/E. canis/H. canis*	1		
Total Number of sentinels with at least one TBP	59	43.4	34.9–52.1

Dog and cat sampling was conducted in both the rainy and dry seasons from both sick and healthy individuals. Table 5 shows the prevalence of each TBP by season and health status. For serology, antibodies against *Anaplasma* and *Ehrlichia* spp. were more prevalent in sick dogs, and antibodies against *Anaplasma* spp. were more prevalent in the dry season while antibodies against *Ehrlichia* spp. were more prevalent in the rainy season. For PCR testing, *A. phagocytophilum, A. platys, B. canis vogelii*, and *H. canis* were more prevalent in the dry season while *E. canis* was more prevalent in the rainy season. *A. platys, B. canis vogeli, E. canis*, and *H. canis* were also more prevalent in sick dogs, but *A. phagocytophilum* was more prevalent in healthy dogs although sample size was smallest for this pathogen.

**Table 5:** Prevalence for each tick-borne pathogen found in blood samples of dogs by season and health status.

**Pathogen**	**Positive samples (Prevalence %, 95% CI)**		**Positive samples (Prevalence %, 95% CI)**	
	**Sick *N =* 43**	**Healthy *N =* 93**	**Dry *N =* 50**	**Rainy *N =* 86**
*Anaplasma phagocytophilum*	2 (5, 0.6–15.8)	6 (7, 2.4–13.5)	5 (10, 3.3–28.8)	3 (3, 0.7–9.6)
*Anaplasma platys*	11 (26, 13.5–41.2)	15 (16, 9.3–25.2)	10 (20, 10.0–33.7)	16 (19, 11.0–28.4)
*Babesia canis vogeli*	6 (14, 5.3–27.9)	6 (7, 2.4–13.5)	6 (12, 4.5–24.3)	6 (7, 2.6–14.6)
*Ehrlichia canis*	6 (14, 5.3–27.9)	12 (13, 6.8–21.5)	5 (10, 3.3–28.8)	13 (15, 8.3–24.5)
*Hepatozoon canis*	7 (16, 6.8–30.7)	13 (14, 7.7–22.7)	8 (16, 7.2–29.1)	12 (14, 7.4–23.1)

Results of a binary logistic regression univariable analysis to determine any relationship between TBP positivity for each of the five pathogens, season, and health status are listed in Tables 6, 7. The likelihood of infection was greater in the dry season for *A. phagocytophilum, A. platys, B. canis vogeli*, and *H. canis*. In contrast, *E. canis*, antibodies against *Ehrlichia* spp., and antibodies against *Anaplasma* spp. indicated that the likelihood of infection was lower in the dry season. The OR for sick health status was greater than one for antibodies against *Anaplasma* spp., *A. platys, B. canis vogeli*, antibodies against *Ehrlichia* spp., *E. canis*, and *H. canis* indicating blood samples were more likely to be positive if the patient was sick. The OR was less than one for *A. phagocytophilum*. Results with statistical significance (*p* < 0.05) were *A. platys* positivity with a greater likelihood of being positive with an *Anaplasma* spp. antibody test; *Anaplasma* spp. antibody test with a greater likelihood of being positive in a sick animal; *Anaplasma* spp. antibody test with a greater likelihood of being positive with a positive molecular test for *A. platys*; and, *Ehrlichia* spp. antibody test with a much greater likelihood of being positive with a positive *E. canis* molecular test.

**Table 6:** Relationship between PCR positivity for each tick–borne pathogen with health^*^ status and season^**^ (univariable analysis).

	***Anaplasma phagocytophilum***		***Anaplasma platys***		***Babesia canis vogeli***		***Ehrlichia canis***		***Hepatozoon canis***	
	**Health**	**Season**	**Health**	**Season**	**Health**	**Season**	**Health**	**Season**	**Health**	**Season**
B	−0.35	1.12	0.58	0.09	0.86	0.60	0.19	−0.38	0.18	0.16
S.E.	0.84	0.75	0.45	0.45	0.61	0.61	0.55	0.57	0.51	0.50
p	0.68	0.14	0.20	0.84	0.16	0.33	0.73	0.50	0.73	0.75
Odds ratio	0.71	3.07	1.79	1.09	2.35	1.82	1.21	0.69	1.20	1.18
95% CI	0.14–3.66	0.70–13.46	0.74–4.31	0.45–2.64	0.71–7.77	0.55–5.98	0.42–3.52	0.23–2.07	0.44–3.25	0.45–3.10

* *Reference is sick.*

** *Reference is dry*.

**Table 7:** Relationship between positivity for antibodies against *Ehrlichi*a spp. or *Anaplasma* spp. with health^*^, season^**^, or corresponding positive PCR test (univariable analysis).

	***Anaplasma*** **spp. antibody**				***Ehrlichia*** **spp. antibody**		
	**Health**	**Season**	***A. phago***	***A. platys***	**Health**	**Season**	***E.canis***
			**PCR**	**PCR**			**PCR**
B	1.28	−0.12	0.28	1.18	0.92	−0.36	1.74
S.E.	0.39	0.39	0.76	0.45	0.49	0.52	0.57
p	0.001	0.76	0.71	0.009	0.061	0.50	0.002
Odds ratio	3.59	0.88	1.32	3.26	2.52	0.70	5.71
95% CI	1.66–7.76	0.42–1.89	0.30–5.80	1.35–7.86	0.96–6.60	0.25–1.96	1.85–17.57

* *Reference is sick.*

** *Reference is dry*.

## Discussion

This study used two different methods to collect ticks in Guam. Environmental sampling was not nearly as fruitful and efficient as animal sampling for ticks. In this study, ticks were successfully recovered using environmental sampling techniques in 63% of events, but only low numbers of one tick species were found. The season, daily weather pattern, and location all contributed to the success of a given sampling event. Thus, it would be difficult to discover a newly introduced species of tick via environmental sampling methods unless the tick species had already become widespread. Interestingly, *R. sanguineus* is usually found closer to urban dwellings (49) but it was widespread in Guam. Although rural areas have more deer, wild pigs, water buffalo, and cattle which host *R. microplus*, large populations of feral and stray dogs may be sufficient to maintain *R. sanguineus* in these rural areas. As with most tick species, there were specific habitats where *R. sanguineus* was detected with environmental sampling. The southern part of Guam, which has large regions of rangeland landcover (savanna complex habitat specifically) was where *R. sanguineus* was most often recovered. This could be because this vegetation type is easier to conduct dragging and flagging compared with rockier and heavily forested areas, or this could be a preferred habitat type for this tick.

The dry season, which includes the months from January through June, was the most common season for recovering *R. sanguineus* from the environment. Although it is called the dry season, rain still falls regularly; however, this will vary from year to year. Flagging and dragging success is dependent on the weather for that day, and every effort was made to only conduct sampling when there was no recent overnight rain. Sampling is difficult in the rain since it was harder to acquire ticks on the cotton flag when it was soaked. The likelihood of finding ticks (positive sampling event) was roughly eight times greater in the dry season compared to the rainy season (*p* = 0.032).

In contrast, collection of ticks from animals was much more successful in regard to numbers of ticks and species. Dogs, wild pigs, and some cats are effective sentinels since they travel long distances and roam in both isolated wildlife habitat and near human dwellings. Animal tick infestations are also not dependent entirely on weather and terrain (37, 50). From these three sentinel species, four different tick species were found. *Rhipicephalus sanguineus* was the most prevalent tick species found, which is unsurprising as it is the most widespread tick in Guam and can live in a variety of habitats from tropical forests to urban dwellings. *Rhipicephalus sanguineus* is predominantly an ectoparasite of the domestic dog and is believed to have been involved in the transmission of pathogens for over 2,000 years (51); however, it can attach and feed on other hosts such as cats, various birds, rodents, and humans (49). The most important concern with *R. sanguineus* is its role as a vector of numerous pathogens (30, 52–56). There are two clades or lineages, temperate and tropical, which are significant in that these lineages are associated with different pathogens (57). In Guam, we only detected the tropical lineage of *R. sanguineus* which is not surprising given how common *E. canis* was in dogs and this lineage is more likely to transmit *E. canis* (58). However, only a limited number of ticks were genotyped so additional work is needed to determine if both lineages are present in Guam.

*Rhipicephalus microplus*, the southern cattle tick, was the second most common tick found. Annual losses due to the impact of this tick species particularly in tropical countries is estimated to be 22–30 billion US dollars per year (59). In Guam, where there is no commercial livestock farming, *R. microplus* ticks are mostly found on Philippine deer, cattle, water buffalo, and goats (24, 27, 29). *Rhipicephalus microplus* is a one-host tick (8) so it was unusual to find this tick on dogs and cats as they are not the typical host; however, several studies have reported finding this tick on dogs, cats, and wild carnivores (60–64). *Rhipicephalus microplus* can host various TBP. Cleveland et al. (27) did find “*Candidatus* Rickettsia senegalensis,” of the *R. felis* cluster, a potential human pathogen, in *R. microplus* collected from Philippine deer in Guam. This pathogen has been reported most in cat fleas (*Ctenocephalides felis orientis*), but other arthropods such as ticks can host “*Candidatus* Rickettsia senegalensis” (65–67).

Several immature specimens of a *Haemaphysalis* sp. were detected which represents the first detection of this genus on Guam. This tick belongs to a genus that includes at least 168 different species. It is a small, inornate tick that has a three-host life cycle. Adults are found in both domestic animals and wildlife while immature stages prefer to parasitize small mammals and birds (68). Immature stages are difficult to identify to species and many species lack sufficient descriptions, so species identification was not conducted; however, they are morphologically and genetically distinct from *H. longicornis*, a species native to East Asia of recent notoriety because it is invasive in the United States (23, 69) and numerous countries in Oceania (70, 71). Assuming this tick has been introduced, where this *Haemaphysalis* sp. originally came from is unknown; however, Guam frequently has travelers, sometimes with companion animals, from East Asia where numerous *Haemaphysalis* spp. are endemic. Another possibility is that a migratory bird could have introduced this species as this genus has been found on migratory birds in Asia (72). With the introduction of a non-native tick species, the risk of a new TBD increases. A concerning finding is that multiple *Haemaphysalis* spp. in native and introduced areas can transmit numerous pathogens (73, 74).

Due to their close association with humans and interactions with wildlife, domestic canines can act as sentinels for vector and/or pathogen exposure for humans and other wildlife species (75, 76). Sampling can be easily done as part of veterinary hospital visits or as part of vaccine and preventive care campaigns in more rural areas (41). Dogs may potentially be a reservoir of certain infectious disease and be a source of infection for ticks (77). For zoonotic pathogens, dogs may serve as effective sentinels (78, 79). For example, seroprevalence of *Borrelia burgdorferi* in dogs may be a sensitive marker of human risk for Lyme disease as dogs are infected at a higher frequency (80, 81).

Numerous TBP were detected in blood samples of dogs and cats including *A. phagocytophilum, A. platy*s, *B. canis vogeli, E. canis*, and *H. canis*. This is the first time these pathogens have been identified molecularly in dogs and one cat in Guam. *Anaplasma platys* is a mostly canine pathogen that is transmitted by *R. sanguineus*. *Anaplasma phagocytophilum* is a known zoonotic agent and infects a wide variety of species to include birds, cats, rodents, and deer species, including Philippine deer in Guam (29, 82). *Babesia* spp. are intraerythrocytic protozoans grouped in the Piroplasmidae family, related to *Theileria* spp. *Babesia canis*, a large piroplasm, infects dogs and is subdivided into subspecies based on unique biology and tick vectors. *Babesia canis vogeli* is transmitted by *R. sanguineus* (83, 84). *Ehrlichia* are gram-negative, obligate intracellular, pleomorphic organisms of the Rickettsia order. *Ehrlichia canis* is the agent of canine monocytic ehrlichiosis (85). *Hepatozoon* is a genus of protozoal parasites which includes over 300 different species. *Hepatozoon canis* is of primary concern for dogs (86) with infections ranging from subclinical and asymptomatic to severe (87). *Anaplasma platys* was the most common TBP found. This is consistent with what has been anecdotally noted in the population of dogs seen at animal hospitals in Guam. The antibody assays used in this study can cross-react with several species within these genera, but there was a statistically significant correlation between antibody results and positive corresponding molecular testing for *E. canis* and *A. platys* suggesting that serologic data corresponds with these two species.

Nearly half of the dogs (43.4%) tested were positive for at least one TBP. Another 14.7% had infections with two or three TBP. Although most pathogens were more likely to be found in the dry season and in sick individuals, none of the relationships were statistically significant. This is consistent with what is seen in the animal hospitals in Guam. Not all animals that are sick with a TBP will test positive, and many animals are positive for a TBP and lack disease. In fact, clinically ill animals often have other comorbidities that may make illness due to TBP opportunistic. A larger sample size might elucidate any relationships present although this may not be important in monitoring prevalence of TBP for a tick monitoring program.

The detection of *A. phagocytophilum* in dogs is an interesting finding as it confirms the recent detection of this pathogen in Philippine deer from Guam (29) despite the typical vector (*Ixodes* spp.) not being detected on these hosts in Guam. Although *A. phagocytophilum* is usually associated with an *Ixodes* spp. (72), there are competent vectors in other tick genera. The most common tick on deer in Guam was *R. microplus* and although this tick was rarely found on dogs in this study, *R. microplus* can transmit *A. phagocytophilum* (88). The predominant ticks on dogs were *R. sanguineus*, and several studies have detected *A. phagocytophilum* in *R. sanguineus* (89–91). In addition, *H. longicornis* in the Republic of Korea (South Korea) (92) and United States (74) were positive for *A. phagocytophilum*. There are also several Asian species of *Haemaphysalis* (e.g., *H. megaspinosa, H. douglasii*, and *H. japonica*) have been PCR positive for *A. phagocytophilum* (72).

*Anaplasma phagocytophilum* has also been reported from some *Amblyomma* spp. (e.g., in *A. flavomaculatum* from various imported African reptile species in Poland) (93) and in *A. cajennense* from dogs in Brazil) (91). In Europe, wild pigs are a host of *A. phagocytophilum* (94); however, *A. phagocytophilum* was not found in wild pigs in Guam in a recent study (27) nor in the current study. Because of the lack of *A. phagocytophilum* in pigs and a lack of *Amblyomma* spp. on deer or dogs, *A. breviscutatum* is not suspected to be a vector of *A. phagocytophilum*. Although wild pigs are a reservoir of other pathogens such as leptospirosis, there is no evidence from this study that wild pigs are a reservoir of TBP in Guam as none were positive for *Babesia* spp. or *A. phagocytophilum*. Nonetheless, in 2015, there was a reported case of infestation of humans by larval *A. breviscutatum* (95) so determining its vector competence for zoonotic pathogens, especially *Rickettsia* spp., is important. A previous study found *R. amblyommatis*, a potentially zoonotic pathogen, from *A. breviscutatum* from wild pigs in Guam (27). Wild pigs are managed by the USDA in Guam, so this is one way to easily monitor this species in Guam.

Several of the TBP that have been found in the current and previous studies in Guam are zoonotic or have the potential to cause illness in humans (*A. phagocytophilum, C. burnetii, R. amblyommatis, and R. felis*). The most significant is *A. phagocytophilum*, which causes human granulocytotropic anaplasmosis (HGA). The other Anaplasmataceae reported in Guam, *A. platys*, may also infect people although its exact role in disease is not completely understood. Maggi et al. (96) reported a symptomatic veterinarian with seizures and migraines who was positive for *A. platys*, but this patient was also infected with *Bartonella henselae* and “*Candidatus* Mycoplasma haematoparvum*.”* There was another report of two symptomatic people in the US who, along with their dog, were infected with *A. platys, E. chaffeensi*s, and *E. ewingii* (97). In Venezuela, there were two case reports of people with symptoms such as headaches, myalgia, joint pain, and fever, and *A. platys* was the only TBP detected in their blood (98). In all cases, the humans lived or worked closely with dogs. *Ehrlichia canis* can also cause illness in humans with similar symptoms to *E. ewingii* or *E. chaffeensis* (99); however, *E. canis* is more likely asymptomatic as noted in a study of blood bank samples where donors had no symptoms of disease but 3.6% of people had molecular and serological evidence of *E. canis* infection (100).

Of note, there were several limitations to this study. The sample size for off-host sampling was small, and only revealed one statistically significant conclusion. A more robust sample size over a longer period and through multiple seasons would have been more informative. Sampling bias may be present as sampling of both animals and the environment was not randomized. It is recommended that the study be expanded upon by having a more structured means of sampling both the island and the animals. Another limitation of this study is that the ticks themselves were not tested for pathogens, and animals were not tested for other TBP such as *Rickettsi*a spp. that have been found in Guam in previous studies.

## Conclusion

Second only to mosquito-borne pathogens, TBP and their associated diseases are a significant threat to humans, domestic animals, and wildlife worldwide. Sentinel surveillance can be a useful tool in helping to understand the risk of disease from TBP in humans and wildlife (36). From this study and previous studies in Guam (27, 29) dogs and deer may be the most useful mammalian sentinels thus far for determining tick species and TBP prevalence on the island. In fact, the current sentinel surveillance discovered a novel tick species in Guam and a high prevalence of TBP in dogs with almost half of all dogs with one or more ticks infected with at least one TBP. Consistent and on-going sentinel surveillance is essential to detect new ticks and novel pathogens in Guam. Other wildlife species, including birds and reptiles, should also be included as sentinel species for future studies to increase the diversity of ticks that may be detected.

Although Guam is a seemingly isolated island in the middle of the Pacific Ocean, it has an outsized influence in this area and perhaps around the world with large numbers of transitory citizens, many tourists, and rotating military and other government personnel. Almost all residents have family members from outside the island to include the USA, Asia, Russia, and other Pacific Islands. Air travel is very frequent in this population to include movement of pets, and consequently, disease movement. Therefore, understanding the disease threats present in Guam is not only important to the current population but also to other parts of the world.

## Data Availability Statement

The raw data supporting the conclusions of this article will be made available by the authors, without undue reservation.

## Ethics Statement

The animal study was reviewed and approved by Royal (Dick) School of Veterinary Studies Veterinary Ethical Review Committee (VERC) of the University of Edinburgh for animal usage in clinical research with VERC Reference number 89.19. Written informed consent was obtained from the owners for the participation of their animals in this study.

## Author Contributions

GW was responsible for conception of the study, manuscript writing and revisions, data analysis, and interpretation of the data. NA was responsible for conceptualization, supervision, and manuscript review and editing. AT was responsible for tick identification and review of the manuscript. KG was responsible for molecular testing and analysis of blood pathogen data and review of the manuscript. MY was responsible for study design, analysis and interpretation of data, and writing of the manuscript. All authors contributed to the article and approved the submitted version.

## Funding

Experiment Crowd Funding Platform for Scientific Research and the Southeastern Cooperative Wildlife Disease Study at the University of Georgia.

## Conflict of Interest

The authors declare that the research was conducted in the absence of any commercial or financial relationships that could be construed as a potential conflict of interest.

## Publisher's Note

All claims expressed in this article are solely those of the authors and do not necessarily represent those of their affiliated organizations, or those of the publisher, the editors and the reviewers. Any product that may be evaluated in this article, or claim that may be made by its manufacturer, is not guaranteed or endorsed by the publisher.

## Abbreviations

TBD: tick-borne disease(s)
TBP: tick-borne pathogen(s).
