# Supplementary material for: Ticks and Tick-Borne Pathogens in Domestic Animals, Wild Pigs, and Off-Host Environmental Sampling in Guam, USA
Source: Front Vet Sci. 2022 Jan 11;8:803424. doi: 10.3389/fvets.2021.803424 (PMC8787080; doi:10.3389/fvets.2021.803424)
Supplement: Supplementary file 1 [file Data_Sheet_1.docx]

**Table 1: Tick-Borne Pathogens (TBP) That Have Been Directly or Indirectly Detected in Animals and/or the Ticks Feeding on Animals From Guam**

| Pathogen | Method | Vector | Host | Reference |
| --- | --- | --- | --- | --- |
| *Anaplasma platys* | PCR tick  PCR blood | *Rhipicephalus sanguineus* | dog  Philippine deer (*Rusa marianna)* | (28,29) |
| *Anaplasma marginale* | serology  PCR blood | *Rhipicephalus microplus* | cow, water buffalo  Philippine deer | (29,101) |
| *Anaplasma phagocytophilum* | PCR blood | *Rhipicephalus microplus,* +/- *Rhipicephalus sanguineus* | Philippine deer | (29) |
| *Babesia bovis* | serology | *Rhipicephalus microplus* | cow | (101) |
| *Babesia bigemina* | serology | *Rhipicephalus microplus* | cow | (101) |
| *Babesia canis vogeli* | PCR tick | *Rhipicephalus sanguineus* | dog | (28) |
| *Coxiella burnetii* | PCR tick,  serology | *Rhipicephalus sanguineus* | dog, goat, cow | (28,101) |
| *Ehrlichia canis* | serology | *Rhipicephalus sanguineus* | dog | Personal observation |
| *Hepatozoon canis* | PCR tick | *Rhipicephalus sanguineus* | dog | (28) |
| *Rickettsia amblyommatis* | PCR tick | *Rhipicephalus microplus,*  *Amblyomma breviscutatum* | Philippine deer  wild pig | (27) |
| *Rickettsia felis* | PCR tick | *Rhipicephalus microplus* | Philippine deer | (27) |

**Table 2: Landcover Classification System in Guam** (42)

| Landcover classification | Subcategories |
| --- | --- |
| Forest | acacia plantation, casuarina thicket, coastal strand, limestone forest, palma brava grove, ravine forest, scrub forest, mangrove swamp forest |
| Rangeland | savanna complex, strand vegetation, other shrub/grass |
| Urban | urban built-up, urban cultivated, agricultural land such as coconut plantations and agricultural field |
| Barren | badland (dry terrain with minimal vegetation), barren, sand beach, bare rocks |
| Water | water, wetland, marshland |

**Table 3: Primers Used in PCR Assays**

| Pathogen | Gene Target | Primers | Amplicon Size | Reference |
| --- | --- | --- | --- | --- |
| *Babesia*/ *Hepatozoon* | 18S rRNA | 5-22F (5’- GTTGATCCTGCCAGTAGT -3’)  1661R (5’- AACCTTGTTACGACTTCTC -3’) | 1655bp | (48) |
| *Ehrlichia canis* | 18S rRNA | 1:  ECC (5’- AGAACGAACGCTGGCGGCAAGCC-3’)  ECB (5’- CGTATTACCGCGGCTGCTGGCA -3’)  2:  ECA (5’- CAATTATTTATAGCCTCTGGCTATAGG-3’)  HE3 (5’- TATAGGTACCGTCATTATCTTCCCTAT -3’) | 400bp | (102) |
| *Anaplasma platys* | 16S rRNA | 1:  ECC (5’- AGAACGAACGCTGGCGGCAAGCC-3’)  ECB (5’- CGTATTACCGCGGCTGCTGGCA -3’)  PLA2 (5’- TTTGTCGTAGCTTGCTATG -3’)  GA1UR (5’- GAGTTTGCCGGGACTTCTTCT -3’) | 400bp | (102) |
| *Anaplasma phagocytophilum* | 16S rRNA | 1:  ECC (5’- AGAACGAACGCTGGCGGCAAGCC-3’)  ECB (5’- CGTATTACCGCGGCTGCTGGCA -3’)  GE9F (5’- AACGGATTATTCTTTATAGCTTGCT  -3’)  GA1UR (5’- GAGTTTGCCGGGACTTCTTCT -3’) | 400bp | (103) |
|  |  |  |  |  |

**References**

1. Duguies M, Nusbaum P, Saville S. *Animal Health Survey for Guam 1999. ADAP Project for Guam, Northern Marianas Islands, Palau, Federated States of Micronesia, and American Samoa.* Honolulu (2000). Available online at: <https://scholarspace.manoa.hawaii.edu/bitstream/10125/34060/2000-25.pdf> (accessed March 14, 2019).
2. Yabsley MJ, McKibben J, Macpherson CN, Cattan PF, Cherry NA, Hegarty BC, et al. Prevalence of *Ehrlichia canis, Anaplasma platys, Babesia canis vogeli, Hepatozoon canis, Bartonella vinsonii berkhoffii, and Rickettsia* spp. in dogs from Grenada. *Vet Parasitol.* (2008) 151:279–85. doi: 10.1016/j.vetpar.2007.11.008
3. Yabsley MJ, Davidson WR, Stallknecht DE, Varela AS, Swift PK, Devos JC, et al. Evidence of tick-borne organisms in mule deer *(Odocoileus hemionus)* from the Western United States. *Vector-Borne Zoonotic Dis.* (2005) 5:351–62. doi: 10.1089/vbz.2005.5.351
